# Applying Classic Social Psychology Principles to Improve Healthcare Teams

**DOI:** 10.15694/mep.2020.000251.2

**Published:** 2021-09-22

**Authors:** Neil E. Grunberg, John E. McManigle, Erin S. Barry

**Affiliations:** 1 Uniformed Services University

**Keywords:** Social Psychology Principles, Healthcare Teams

## Abstract

This article was migrated. The article was marked as recommended.

Modern healthcare involves teams composed of educators, learners, healthcare providers, patients, patients’ significant others and families, healthcare administrators, and information sources. Principles of social psychology are relevant to interactions among team members when learning and performing professional duties; communicating to build trust, commitment, and teamwork; and collaborating among members of the healthcare team. This paper briefly discusses several classic principles of social psychology and how they apply to healthcare teams and medical education. Understanding and applying these principles will help healthcare providers optimize performance and interactions with colleagues, learners, and patients. It is important to incorporate study and practice of these principles into medical education and professional development.

## Introduction

Modern healthcare involves “teams” - individuals with complementary knowledge and skills working interdependently to achieve common goals ( Katzenbach and Smith, 1999; Hackman, 2002). These teams are composed of educators, learners, many different healthcare providers (physicians, nurses, dentists, psychologists, physical and occupational therapists, social workers, case managers, etc.), patients, patients’ significant others and families, healthcare administrators, and information sources (via computer, Internet, artificial intelligence). Therefore, it is necessary for members of the healthcare team to develop knowledge and skills to optimize interactions and effectiveness with other members of the team.

In addition to the professional knowledge and skills each member of the healthcare team learns, principles of social psychology can be learned and applied to improve healthcare teams as well as medical education. Social psychology is the study of behaviors, cognitions, motivations and emotions in response to the actual, imagined, or implied presence of others ( Grunberg, McManigle and Barry, 2020). Principles of social psychology are relevant to interactions among team members to build trust, commitment, and teamwork ( Gordon, 2013).

This paper briefly presents several, classic social psychology principles to consider for inclusion in medical education to help healthcare providers optimize performance and interactions with colleagues, learners, and patients. Each principle is summarized with background information in roughly chronological order as they first appeared in the academic literature. Then, how each major principle is applicable to healthcare teams and medical education is discussed.

## Classic Social Psychology Principles

### Social Facilitation

*Principle.* Social facilitation refers to presence of people increasing an individual’s “dominant” responses (i.e., easy or well-learned behaviors). If the dominant response is to perform correctly, then performance improves when others are present. If the dominant response is to perform incorrectly (e.g., a difficult task or learning something new), then performance worsens when others are present.

*Background.* Triplett (1897-8) observed that bicycle races with simultaneous competitors were faster than races without simultaneous competitors. He considered various explanations for these performance differences and proposed the social psychological explanation of “social facilitation” - wherein a competitive instinct was aroused in the presence of other competitors. Subsequent research further examined effects of presence of others on performance ( Dashiell, 1930; Henchy and Glass, 1968; Paulus and Murdoch, 1971). Zajonc and colleagues ( Zajonc, 1965; Zajonc and Sales, 1966) reviewed these studies and concluded that the likelihood of a given behavior occurring in the presence of other people depends on whether a given response is strongly developed or dominant (i.e., a response that is most likely to occur). Further, the presence of others increases stress which increases the likelihood of dominant behaviors.

*Applying principle to teams.* Social facilitation is operating whenever others are present, especially in stressful situations and when evaluation is involved. As a result, dominant behaviors increase in these stressful situations where poor interactions among members of the healthcare team can have serious consequences. Members of healthcare teams evaluate themselves and each other’s knowledge and performance, and patients evaluate their providers. Therefore, it is important to train as teams as much as possible (to bring forward the expected dominant responses) and find ways to reduce stress wherever possible.

Additionally, social facilitation is relevant when framing feedback to members of a healthcare team to minimize stress during initial training. Similarly, settings and style to provide medical information to patients should consider social facilitation. Dominant response of learners and patients ranges from calm, focused attention to inattention, anxiety, and catastrophizing. Healthcare professionals should be aware of their own dominant styles as well as styles of learners, patients, family members, and significant others in order to optimize interpersonal connections, learning, and understanding of healthcare options.

*Applying the principle to medical education.* Clinical preceptors evaluate medical students, interns, residents, and fellows. Therefore, dominant behaviors increase in evaluative settings, whether in the classroom, clinic, or bedside. To optimize performance, stress (including physical, psychological, and situational stress including time constraints, evaluations) should be minimized when learning a task and encountering new challenges and situations, especially if the situation is difficult and errors are likely. Because members of the healthcare team often perform under stress, it is important that correct behaviors become dominant responses to optimize success. If the team or its members are inexperienced or if likelihood of errors is high (e.g., because the task is difficult or the case is complex), then it is important to attenuate stress. Once correct responses are well-learned, simulations and practice with stress imposed (e.g., time constraints, limited resources, more critical feedback) are valuable to learners.

### Group Dynamics

*Principle.* Group dynamics refers to influences of groups on individual members’ behavior and cognitions more broadly than social facilitation *per se.* Groups are particularly influential on individuals who consider themselves to be members of the group or who feel compelled to align with the group.

*Background.* Group dynamics involve social norms, group influences, group performance, and group cohesiveness. Sherif (1935) focused on the influence of social norms on perception of physical reality, revealing that salient and valued social groups have an impact on reports and even perceptions about the physical world. Newcomb (1943) studied competing influences of family, college climates, and reference groups on individuals’ beliefs and behaviors. He noted the powerful, short-term influence of salient social groups versus the long-term influence of early life exposures and deep long-lasting relationships.

*Applying principle to teams.* Group dynamics are relevant to the extent of influence of all members of the healthcare team on each other. Social influences are powerful, especially when they are salient and when acceptance by others (including educators, colleagues, patients) is valued. Individuals who are particularly respected (e.g., a well-established physician), confident, extraverted, or forceful often have the greatest influence. If an individual or members of a healthcare team want to encourage expression of diverse opinions and behaviors, then that desire must be clearly established in a psychological safe environment (i.e., where expression of various opinions and behaviors are encouraged and not punished). Individuals who are most respected, relevant, available, and salient will have the greatest influence on opinions, values, and behaviors. Therefore, it is important within team settings to create that safe environment and to be cognizant of the individuals present. You must be aware of your own influence over others and also others’ influences on the members of the team. If certain individuals are dominating the conversation, then you may need to pull team members who are quieter aside to gain their opinions.

In addition, principles of group dynamics indicate that the perceived authority of an individual as well as the perception whether a given opinion is held by the group affect the opinions and behaviors of each person present. For example, in discussing diagnosis and treatment for a given case, whether individual and potentially opposing opinions are discouraged or encouraged will affect the perceptions and responses of the members of the team. Similarly, group dynamics influence patients, significant others, and their families with regard to decision-making relevant to healthcare prevention and treatment. Whether and how relevant others’ opinions, attitudes, and behaviors are framed and become salient will affect decisions and behaviors of patients and whether the behaviors are consistent with best healthcare practice.

*Applying principle to medical education.* Educators and more experienced healthcare professionals must realize that their reactions, verbally and non-verbally, have powerful effects on whether opinions of less experienced colleagues and learners are even expressed. In learning settings, educators should be aware of their influence on learners. Educators must be sure when asking questions (e.g., “Should this patient be put on omeprazole?”) that learners fully understand and do not just hear what the educator or others want to hear (e.g., “Yes, I’ll take care of that.”). It is important as an educator to create a safe learning environment so that learners are able to ask their questions and learn and not be overly constrained by the educator’s influence over them or the other team members’ opinions.

### Field Theory

*Principle.* Field Theory in Social Science (or “field theory”) describes each individual’s behaviors, cognitions, and motivations as one’s “Life Space” with personal goals identified either as desirable and sought (“positive”) or undesirable and avoided ("negative"). Field theory also describes how each person’s “life space” relates to those of other people including elements of group dynamics.

*Background.* Lewin (1936; Lewin, 1951) believed that mathematizing individuals’ behaviors, cognitions, motivations as well as interactions among individuals would help to understand individuals’ current psychology, predict future psychology, and explain interactions among people. Field theory was based on the notion that behavior is a function of each person and the physical, psychological (intrapersonal), and social (interpersonal) environments. Each individual’s “life space” includes the psychological “location” of the person with regard to behaviors, cognitions, motivations, and forces acting on each person (intrapersonally and interpersonally).

*Applying principle to teams.* The life spaces of members of the healthcare team are represented as overlapping life spaces ( Yarnell and Grunberg, 2017). If the positive and/or negative valence goal regions of the interacting individuals are aligned or congruent, then those individuals will cooperate, work together, and have positive relationships. If goal regions with similar valence are not aligned between individuals, then those individuals will struggle to form cohesive or cooperative teams. Within each of our life spaces, we are drawn to and hope to attain positive goal regions, such as good health, happiness, meaningful relationships, professional and personal success. We try to avoid and move away from (in actions and thoughts) negative goal regions, such as illnesses, injuries, failures in our personal and professional lives. Our life spaces overlap with people with whom we interact (including colleagues, family members, friends, patients). Therefore, aligning positive and negative goal regions among people with whom we interact regularly will increase cohesiveness, coordination of efforts, and shared successes. It is often helpful to find those goal regions that we “share” with others to optimize performance, cohesiveness, and morale. Therefore, members of the healthcare team should communicate emotions with each other so that positive alignment can occur towards similar goal regions. Being aware of your own and others’ negative goal regions also can allow teams to have more effective interactions with members of the healthcare team by increasing understanding, morale, and compliance.

*Applying principle to medical education.* Similar to teams, it is important for educators to be aware of their own life space as well as those of their learners. Without this awareness, educators may inadvertently provide feedback in a way that evokes negative emotions or responses in learners. It is particularly important for educators to be aware of learners’ positive and negative goal regions as well as where the learners are within their own life spaces. Educators should work to ensure that positive goal regions of learners are well aligned with the educator’s desired goal for the learners. In addition, educators should do whatever they can to diminish or attenuate negative goal regions of learners that might interfere with the learners’ progress. This could be done by highlighting the value of the positive goal region and helping learners to cope with and surmount negative goal regions that may detract from their progress. Further, understanding where learners are in their life spaces relative to the educational goals will assist the educator in meeting the learners wherever they are.

### Informal Social Communication

*Principle.* Informal social communication holds that unplanned interactions and communication with other people influences opinions, attitudes, beliefs, and behaviors and that “functional distance” (i.e., interactions with other) is more important that “physical distance” (the actual amount of geometric space).

*Background.* After World War II, large numbers of veterans furthered their educations and universities created make-shift student housing in the United States. Festinger, Schachter, and Back (1950) realized that different housing designs affected unplanned interactions students had with each other. They reasoned that the more frequently people interact, the more they are likely to influence each other’s attitudes and opinions and become cohesiveness groups. Frequency of interactions is related to housing design, staircases, walkways, courtyards, and so on, that affect likelihood of interactions. The more interactions that occurred, the more similar became attitudes and opinions among individuals who interacted. These findings influenced architectural design of college dormitories (e.g., suites of several bedrooms and a common area vs. individual rooms); suburban neighborhoods (e.g., cul-de-sacs and convenient small parks and play areas); and work spaces (e.g., open area work stations with group areas for work and for breaks). Although the original research in informal social communication addressed in-person interactions, virtual interactions including video contact and social media can have similar effects. In fact, social media and current algorithms that powerfully influence message exposure are taking full advantage of information social communication theory.

*Applying principle to teams.* Informal social communication reveals the power of frequent interactions to increase opinions, attitudes, behaviors, and social cohesion. Appropriate design of functional space (physically and virtually) goes a long way to maximize this phenomenon that can have a powerful effect on informal social communication. Shared, attractive break rooms; individual work spaces that allow privacy as needed but which also encourage comfortable interactions; passageway design that increases opportunities to interact with other team members; and readily accessible virtual chat rooms all contribute to interactions and informal social communication. Design of examination, treatment, and meeting rooms (including physician offices) also should consider this principle. For example, the number of chairs, their orientation in each of these spaces, and their relative heights affect interactions of healthcare providers, patients, and others who are present.

*Applying principle to medical education.* It is important to determine and clearly communicate where, when, and how educators and learners should interact. Increased access and use of virtual interactions should consider and establish appropriate ways and times for educators and learners to effectively implement informal social communication to optimize learning. It is useful to realize that frequency of contact can be highly influential. Therefore, educators should be well aware of the frequency of exposure to key messages and information that they deem important for the learners to know and master. Educators can provide relevant lessons either in person, via distance learners, or by encouraging learners to exchange the key messages when speaking with each other in person or in virtual discussions.

### Deindividuation and Individuation

*Principle.* Deindividuation refers to the psychological “need” to be part of a group, whereas Individuation refers to the psychological “need” to be singled out and recognized.

*Background.* Festinger, Pepitone, and Newcomb (1952) proposed that all individuals have particular needs (e.g., food to satisfy hunger; fluids to satisfy thirst) and “quasi-needs” (psychological conditions that must be satisfied). Among these quasi-needs are deindividuation and individuation. Deindividuation is the need to become an unidentified member of a group. Individuation is the need to be recognized, identified, valued as an individual by others and distinct from a group. A group is more influential and desirable if it satisfies both types of need for each member of the group. Military and similar, regimented organizations are particularly good at providing both deindividuation (with standard clothing, haircuts, behaviors) and individuation (with awards and recognition ceremonies).

*Applying principle to teams.* Opportunities to satisfy both types of need increase the healthcare team’s attractiveness and cohesiveness. When individuals are deindividuated (based on similar clothing, masks that hide identities, or when lighting is dim or dark), self-awareness decreases, sense of personal responsibility decreases, and likelihood of engaging in otherwise restrained behaviors increases. The uninhibited behaviors can be risky or harmful to others, or they can be playful and enjoyable for all involved. The consequences of deindividuation depend on the group and situational context. Individual roles were previously distinct and easily identified based on clothing and styles of interactions. That is no longer true. Today, overlapping roles, wearing similar clothing, wearing protective face masks, and identifying as members of a healthcare team enhance deindividuation. Providing individuals within a given subgroup (e.g., learners, fellows, attending physicians) opportunities to connect and identify with each other strengthens deindividuation.

It also is important that individuals are individuated within the team (e.g., with name tags, distinctive face masks, and, possibly, roles indicated). Meaningful contributions, special days, individual successes also should be recognized and applauded. Similarly, these principles can be used to help patients. For example, understanding that a medical condition is shared by others can be valuable, especially when there are physical and behavioral health challenges. Knowing that others have faced, dealt with, and hopefully overcome these challenges provides valuable social support. Recognizing each person as an individual with distinctive experiences and challenges also is a must.

*Applying principle to medical education.* Educators should realize that learners need to be both deindividuated and individuated for optimal psychological satisfaction. Deindividuation is highlighted when groups of learners are addressed in ways that emphasize that they are, indeed, a group of comparable others or peers. Deindividuation can be used effectively to create psychological safety when addressing topics that might otherwise be uncomfortable for the learners. Individuation should be used to recognize contributions, comments, and/or questions of individual learners that the educator wants to particularly reward.

### Social Comparison

*Principle.* According to social comparison theory, people have a need to judge the “goodness” of their behaviors and cognitions. In the absence of physical realities or clear indications of correctness, people rely upon comparisons to similar people and upon the behaviors and opinions of people whom they admire.

*Background.* Festinger (1954) proposed that people need information upon which to judge or evaluate their own behaviors and cognitions (perceptions, attitudes, beliefs, opinions). When a physical reality is available (e.g., Can I lift this box? Can I jump over this stream?), then people are satisfied. When a physical reality is not available, then people use “social reality” to evaluate their cognitions and behaviors. Social reality comes from comparison with similar others and with people who we admire. If one wants to feel particularly good about oneself, then we engage in “downward comparison” by comparison to people who do not know as much or who do not perform as well on the task. If one wants to improve, then we may engage in “upward comparison” by comparison to people who are more knowledgeable or more skilled.

*Applying principle to teams.* Social comparison is a powerful phenomenon that is broadly relevant to interactions among people. When team members are perceived as “comparable” others, then they are readily available for social comparison. The opinions, attitudes, and behaviors of respected members of the healthcare team are particularly influential when right vs. wrong, correct vs. incorrect, legal vs. illegal are not absolute, which may occur when making healthcare decisions.

Upward social comparison occurs when an individual wants to improve and when an individual recognizes the value of looking towards someone(s) who is more knowledgeable, experienced, or skilled to make a given decision (e.g., a senior, respected colleague, supervisor, preceptor). Upward comparison also occurs when patients look to healthcare professionals for guidance about which option to select. Patients will turn to similar others (e.g., other patients in similar situations), including physical and behavioral health conditions, to evaluate their own conditions and what treatments to follow.

*Applying principle to medical education.* Social comparison is maximally operative in the absence of physical realities. Because behavior is powerfully affected by social comparison, educators should emphasize and make desired behaviors of learners most salient (e.g., coming on time to sessions, being well prepared, interacting respectfully with each other). Undesired behaviors of learners should be pointed out as inconsistent with the behaviors of comparable others (e.g., peers, excellent learners). Educators can help shape desired behaviors in learners by remembering principles of social comparison.

### Conformity

*Principle.* Conformity refers to the tendency of individuals to adopt beliefs and behaviors of other people with whom they interact. Conformity is most likely when there is little diversity of perspectives, opinions, and behaviors, and when it is not “safe” to not conform.

*Background.* Asch (1966) studied conformity using perception tasks (e.g., length of a line) that had correct and incorrect answers based on physical reality. Many people reported demonstrably incorrect judgments and answers to conform with other members of a group. In some cases, reports coincided with altered perceptions of physical reality; in other cases, reports resulted from uncertainty or desires to conform. An individual who perceives that an opinion differs from an entire group is likely to conform to the group’s opinion, whereas an individual with access to even one other person who shares their opinion is less likely to conform to the group. Conformity to a group increases as desire to be a member of the group increases. This “Asch effect” depends on the type of information (e.g., the extent to which there is a confirmable physical reality), experience of the individuals involved, size of the group, and confidence in one’s opinion.

*Applying principle to teams.* The Asch effect can be powerful and undermine diverse perspectives and opinions. How expression of opinions is framed and how opinions are offered (e.g., first writing down opinions vs. first sharing out loud with the team) can alter the extent of conformity and the extent of comfort expressing diverse opinions ( Nembhard and Edmondson, 2006). The Asch effect can influence likelihood of medical errors. For example, if an individual healthcare team member notices what may be a problem (e.g., misreading the units on a prescribed medication), it is critical that possible errors are brought up for careful consideration, rather than going along with the explicit or tacit position of the group. Similarly, if an individual holds a different opinion about a diagnosis or treatment strategy, it is important that the social context is such that the individual is encouraged to express that opinion for careful consideration.

The same principle applies for patients and their advocates. That is, patients are likely to “go along” with what they perceive to be everyone else’s opinion (including opinions of healthcare team members and patient’s family and significant others). Therefore, it is important that members of the healthcare team create a safe environment so that the patient feels able and willing to ask any questions and raise any concerns them may have with the healthcare team.

*Applying principle to medical education.* Individuals who have less experience, authority, and/or confidence, such as learners and novice healthcare professionals, are especially prone to the Asch effect and going along with their perception of the opinions of the group. It is important to establish a safe environment where learners and less experienced professionals are encouraged to raise their questions and concerns.

### Balance Theory and Cognitive Dissonance

*Principle.* Balance theory holds that people prefer consistency among people who we like or dislike. Cognitive dissonance theory similarly holds that people are most comfortable when behaviors and beliefs are consistent or “consonant” and are uncomfortable when behaviors and/or cognitions are inconsistent or "dissonant."People tend to change their minds or behaviors to achieve consonance.

*Background.* Cognitive dissonance theory postulates that people are uncomfortable when they have engaged in inconsistent behaviors, have inconsistent cognitions (e.g., perceptions, beliefs, attitudes, ideas), or have behaviors and cognitions that are inconsistent with each other ( Festinger, 1957). When one is self-aware that these inconsistencies exist, then cognitive dissonance results. Festinger’s notion about psychological discomfort from dissonant thoughts and/or behaviors was inspired by Heider’s work (Heider, 1946, 1958) on balance theory - that people prefer “balance” or consistency in their perceptions, attitudes, and judgments. Balance and consonance of attitudes and/or behaviors is the desired psychological state; dissonance of attitudes and/or behaviors is the undesired state.

*Applying principle to teams.* Because cognitive dissonance is psychologically uncomfortable, people tend to change cognitions and/or behaviors to achieve consonance. This phenomenon affects what we think, do, and report. People particularly want to have consonance with others who they value, such as team members and respected leaders. It is relevant to recognize the tendency to alter cognitions and behaviors to be consistent within individuals and with perceptions of valued others’ cognitions and behaviors. When a patient is answering questions for a medical history, is the reported information correct or is it altered to achieve consonance with the patient’s perception of the healthcare provider’s preferences? For example, unless “permission” is given for dissonance, patients are more likely to report behaviors that are not quite accurate (e.g., regarding cigarette smoking, alcohol use, diet, exercise, medication compliance). It is vital to establish rapport and a non-judgmental environment to find out the truth without it being shaded purposely or unconsciously by patients to achieve psychological consonance.

*Applying principle to medical education.* It is important for medical educators to be aware of the discomfort experienced by learners who perceive that they are in a cognitively dissonant state. To optimize learning, educators should work to establish and maintain interactions that permit for “mismatches” among opinions, beliefs, and attitudes. That is, conflicting cognitions (opinions, beliefs, and attitudes) need to be allowed in a safe and respectful psychological environment to encourage learners to express their opinions, ideas, and questions and to allow educators to shepherd them to cognitions and behaviors aligned with best medical practice.

### Affiliation

*Principle.* The principle of affiliation holds that people prefer to be with other people, especially with others perceived to be similar or in similar situations.

*Background.* Schachter (1959) applied social comparison theory to help explain why people affiliate with other people in addition to utilitarian reasons (e.g., for mutual protection; to engage in work that requires more than person) and biological reasons (e.g., to engage in physical contact and sexual relations). Schachter proposed that people also affiliate to compare behaviors, cognitions, and motivations/emotions to similar others in many situations, especially when they are uncertain or under stress ( Schachter, 1959). He argued that we affiliate with people who we perceive to share our life experiences and who will understand us and the situations we face. Or: “misery does not love company; misery loves miserable company.”

*Applying principle to teams.* We are drawn to and find solace from other people who we perceive to share our life experiences and challenges, especially when we are under stress. That stress includes physical stress (e.g., tired, hungry, cold, hot), mental stress (e.g., anxiety, depression, worry), situational stress (e.g., danger, threats, failures, time limitations), and economic stress (e.g., lack of funds). Teams are particularly attractive when the members share life experiences and perspectives. Healthcare professionals at all levels of training and experience can be best supported and avoid burn out by being with, sharing concerns, and identifying coping strategies with other healthcare professionals who are in similar situations (including professional, personal, and family challenges). Similarly, patients who share physical (e.g., cancers, cardiovascular diseases, diabetes, multiple sclerosis) and mental (e.g., anxiety, depression, grief) health conditions can provide valuable support for similar others. In addition, healthcare providers should be sensitive to patients’ perceptions, values, and affiliation needs.

*Applying principle to medical education.* It is valuable to provide opportunities for learners to affiliate with each other to share ideas, experiences, and concerns. Affiliation can be in-person gatherings or it can be achieved virtually. When physical or virtual gathering occurs with comparable others, it is likely that the participants will experience social support and reduced stress. Similarly, educators should find ways to affiliate with other educators to learn from each other, experience social support, and minimize stress.

### Persuasive Communication and Attitude Formation

*Principle.* There are specific communication strategies and techniques that are particularly persuasive and which can shape the attitudes of other people.

*Background.* Social psychologists have a great deal of interest in communication (sending and receiving, verbally and non-verbally) because it is central to human relationships. Persuasive communication influences opinions, behaviors, team building, performance, learning, and cohesiveness. Key principles include: authenticity ( Pearce, 2013); clarity ( Marshall, Harrison and Flanagan, 2009); consistent verbal and nonverbal communication ( Imada and Hakel, 1977; Mehrabian, 2017); point/counterpoint ( Lumsdaine and Janis, 1953); resistance to “counterpropaganda” ( Lumsdaine and Janis, 1953); recency and primacy of messaging ( Miller and Campbell, 1959); perceived self-interest ( Wiener, LaForge and Goolsby, 1990); perceived source credibility on communication effectiveness ( Hovland and Weiss, 1951; Walster, Aronson and Abrahams, 1966); power of repetition ( Weaver *et al.*, 2007).

*Applying principle to teams.* Communication is how individuals influence each other, whether information is explicitly stated, implied, or displayed non-verbally to model or mimic. Healthcare professionals have instruments (ranging from stethoscopes to fMRI scanners; band aids to scalpels), medications (non-prescription and prescription), and communication (verbal and non-verbal) to influence the lives of patients. It is important to recognize and master elements of effective communication - including sending and receiving (especially listening) - to optimize team performance, reduce or eliminate medical errors, and promote health and well-being among patients. It also is important to recognize that styles and elements of communication have profound effects on relationships among colleagues and can either enhance cohesiveness, cooperation, and morale, or can undermine team work.

*Applying principle to medical education.* Communication is central to education. Therefore, educators need to be aware of the principles of effective communication to clearly convey the material in ways that the learners will understand. In addition, learners should be taught the principles of effective communication to help them become outstanding professionals who can both send and receive information, verbally and non-verbally, in ways that optimize understanding with patients and with colleagues.

### Attribution and Misattribution

*Principal.* Attribution theory addresses people’s interpretation of causes that are most influential in various situations. Misattribution is the incorrect understanding of cause, including purposeful manipulation of presumed cause.

*Background.* How we perceive and attribute (or misattribute) cause and effect are aspects of psychological reality relevant to views of self and others and, therefore, impact relationships and teams ( Shaver, 1975). When our perceptions of cause and effect of cognitions and behaviors of self and others match, we exist in similar psychological realities. However, that is not always true. The “fundamental attribution error” is the common phenomenon that: (a) when things go well for us, we attribute the cause to our own doing, whereas when things go poorly for us, we attribute cause to external factors, and (b) we tend to reverse causality when considering the successes and failures of other people ( Ross, 1977). Misattribution ( Storms and Nisbett, 1970) refers to an incorrect perception and designation of cause (e.g., I could not sleep last night because of indigestion; rather than I could not sleep last night because I was worrying).

*Applying principle to teams.* “Leader attribution error” is the phenomenon of giving too much credit or too much blame to the leader, when the success or failure was mostly due to the team members. This type of attribution error can undermine the team members’ sense of responsibility and efforts. To build trust among members of the healthcare team, the leader should take responsibility for any team failures and share the credit when the team succeeds.

Attribution and misattribution also can contribute to patient experiences and responses to treatments. For example, when a child is about to receive a vaccination, the healthcare provider who says, “This isn’t going to hurt” creates an expectation that is usually disconfirmed, thereby undermining trust, credibility, and future expectations. In contrast, the provider who says, “This is going to hurt for a few seconds and then the hurt will stop quickly,” sets up an entirely different expectation. Another example of attribution and misattribution can be applied to insomnia medications. If a patient expects the medication to work immediately to help fall asleep, then the fact that some time passes before there is any induction of sleep is often accompanied by increased alertness and worry, thereby reducing the effectiveness of the medication. Conversely, if desirable expectations of medication effectiveness for a condition in which patients are unaware of actual bodily state (e.g., blood pressure) are encouraged, then the attribution can enhance the medication’s effectiveness as it adds a placebo effect to the actual biomedical action.

*Applying principle to medical education.* Learners must be made aware of the tendency for the fundamental attribution error; that is, they need to know when to take responsibility and to be accountable for their own behaviors and cognitions. A realistic self-appraisal is necessary for optimal professional growth and development. Similarly, educators should be aware of and avoid the fundamental attribution error when reflecting upon their performance as teachers. In addition, it is important to recognize that educators also serve as leaders to their learners. Therefore, awareness of the leader attribution error can help individuals recognize the contributions of others.

### Cooperation, Competition, and Conflict

*Principle.* People interact in ways that may be cooperative, competitive, and/or in conflict. These various types of interaction may be positive or negative depending on the situation, the individuals involved, and how the situations and interactions are framed and perceived.

*Background.* Deutsch and colleagues ( Coleman, Deutsch and Marcus, 2014; Deutsch, 2014) studied cooperation, competition, conflict, and conflict resolution. Cooperation involves individuals working interdependently or independently to achieve common or individual goals. Cooperation is usually considered to be positive. However, cooperation can be negative when individuals make or perceive they make markedly different contributions. Competition involves individuals working to achieve similar goals wherein only one or a few succeed. Competition is considered to be negative if there are clear losers who suffer psychologically and/or physically. In contrast, competition is positive if it motivates individuals to optimize development and performance. The framing of cooperation and competition is key to the positive or negative social interaction experience. Conflict is usually a negative situation when it involves winner(s) versus loser(s) and the consequences are serious. Conflict is positive and valuable when it involves expression of contrasting views to solve a problem.

*Applying principle to teams.* It is critical that everyone understands the purpose of different social interactions and what is appropriate. Framing of conflict can have a profound effect on the behaviors and relationships of the members of a team. Differences of opinion contribute to conflict, but if the conflict occurs with mutual respect and openness to opposing views, then it is a positive situation that contributes to meaningful decision-making.

Members of a sports team often get the most out of practice when there is some competition among teammates, but cooperation is vital to successfully compete with other teams. Musicians and actors usually perform optimally when competing for a position or role, but cooperation is essential for ensemble performances. Members of interprofessional healthcare teams often bring a pride and esprit de corps from their individual profession or specialty, but they all recognize that effective medicine is a team activity that requires coordination and cooperation. If competition and cooperation are both framed and accepted as ways to strengthen the healthcare team, then both interpersonal processes are positive. If competition is framed as a zero-sum game with winners and losers; or cooperation allows some team members to be slackers while other carry the burden of the task; or conflict is acrimonious and reduces exchange of ideas, then the situations and interactions among members of the team are negative. How members of the healthcare team perceive and engage with regard to these principles will have marked effects on the extent to which ideas are offered and how pleasant/unpleasant, successful/unsuccessful, supportive/unsupportive a team is. Similarly, how interactions are framed with patients in this regard will contribute to the relationship among the healthcare providers, patient, and relevant others for the understanding and well-being of the patient.

*Applying principle to medical education.* Educators need to be aware that the way they frame interactions among and with learners can make best use of phenomena of cooperation and competition. When learners are required to work together on a given task or problem, the value of cooperation can be highlighted by requiring input from each and every member of that team. Competition can be framed in a way that maximizes excellence when done in a way that creates “friendly” competition; that is, a winning team or individual is rewarded but not to the detriment of the competitors. In contrast, situations where non-winners are framed as “losers” in a negative manner can undermine learner morale. Similarly, when conflict is framed in a manner that encourages expression of diverse viewpoints and actions to address or solve a given problem or situation, it is a powerful and effective way to educate and learn from each other. When conflict becomes negative, ad hominem, abusive, unpleasant, it can undermine effective education.

## Summary

Understanding and applying these principles of social psychology will allow members of the healthcare team and medical educators to optimize performance and interactions with colleagues, learners, and patients. It is important to incorporate study and practice of these principles into healthcare education and professional development. The present paper is intended to provide an introduction to some major social psychological principles relevant to healthcare teams and medical education.

## Take Home Messages

• Classic principles in social psychology provide valuable information relevant to interpersonal relationships and teams
• These principles are relevant to healthcare teams, including educators, learners, healthcare providers, patients, patients’ significant others and families, healthcare administrators, and information sources
• This information should be incorporated in medical education programs
• Understanding and application of classic principles of social psychology is likely to optimize healthcare professionals as educators and practitioners

## Notes On Contributors

**Neil E. Grunberg**, PhD, is a Professor in Military & Emergency Medicine (MEM) and Uniformed Services University (USU) Leader and Leadership Education and Development (LEAD) Director of Research & Development. ORCID ID: https://orcid.org/0000-0002-2334-600X

**John E. McManigle**, MD, is Deputy Dean, Senior Advisor MEM, and the USU LEAD Director of Curriculum.

**Erin S. Barry**, MS, is a Research Assistant Professor in the Department of MEM, Research Associate for LEAD, and doctoral student in the Health Professions Education Program, USU. ORCID ID: https://orcid.org/0000-0003-0788-7153
