# Supplementary material for: Applying Classic Social Psychology Principles to Improve Healthcare Teams
Source: MedEdPublish (2016). 2021 Sep 22;9:251. Originally published 2020 Nov 10. [Version 2] doi: 10.15694/mep.2020.000251.2 (PMC10697509; doi:10.15694/mep.2020.000251.2)
Supplement: Peer review history

# Reviewer report: Reviewer response for version 2

**DOI:** 10.21956/mep.20280.r31486

**Published:** 2021-10-09

**Authors:** Madalena Folque Patricio

**Affiliations:** 1 School of Medicine, University of Lisbon

This review has been migrated. The reviewer awarded 4 stars out of 5 This is an useful article because, as the authors said, 'understanding and applying the principles of social psychology will allow members of the healthcare team and medical educators to optimize performance and interactions with colleagues, learners, and patients'. Although I have not read the previous version, I have no doubt that the reinforcement of the application of social psychology principles to medical education reported by the authors, can only have improved this article. My comments go exactly in this direction, challenging the authors to go further. This because the description of the 11 principles is perhaps too extensive when compared to the description of their application to medical education, and not always very clearIt would be important to summarize in a short sentence one 'practical Implication' per principle. It could be something as exemplified below, regarding the first 3 principles:• Practical implication of ‘Social Facilitation’:Educators must optimize performance by reducing learners' stress when learning a task and encountering new challenges and situations, especially if the situation is difficult and errors are likely.• Practical implication of ‘Group Dynamics’:Educators must create a safe learning environment so learners are able to ask their questions and learn from there• Practical implication of 'Field theory':Educators must be aware of their own life space as well as those of their learners because without this awareness, educators may inadvertently provide feedback in a way that evokes negative emotions or responses in learnersThen, the eleven 'Practical Implications' identified, should be reported in the 'Summary'. As several Social Psychology Principles point to the same 'practical implications, the next step should be to bring them together to identify those 3 or 4 common implications and present them as ‘Take Home Messages’.

Reviewer Expertise:

NA

No decision status is available

# Reviewer report: Reviewer response for version 2

**DOI:** 10.21956/mep.20280.r31485

**Published:** 2021-09-29

**Authors:** Ken Masters

**Affiliations:** 1 Sultan Qaboos University

This review has been migrated. The reviewer awarded 4 stars out of 5 I am pleased to see that the authors have located their paper more directly in the field of medical education, and have improved the value of the paper to medical educators. The paper is a worthwhile read for all involved in using small groups and teams in medical education.

Reviewer Expertise:

NA

No decision status is available

# Reviewer report: Reviewer response for version 1

**DOI:** 10.21956/mep.19011.r27280

**Published:** 2021-01-20

**Authors:** Ken Masters

**Affiliations:** 1 Sultan Qaboos University

This review has been migrated. The reviewer awarded 3 stars out of 5 An interesting paper giving advice on applying classic social psychology principles to improve healthcare teams. The article is well-written. It gives much-needed insight into the theory of team interactions, and does it in a way that is grounded in the literature, supplies the correct terminology, and does not over-burden the non-specialist with impenetrable jargon. In doing so, it takes the reader beyond the standard (clichéd) reduction of team dynamics of “Forming, Storming, Norming…etc.”For each principle, it gives a straight-forward explanation, and then the theoretical background, followed by practical advice on how to use this information in healthcare teams. A central weakness of the paper, however, is that it is aimed at healthcare professionals, and so would be useful in a purely medical journal. As this journal is a medical education journal, I would like to see examples applying to medical education. (The current examples could remain, but examples specifically relating to medical education really are required. Obvious places would be complications in PBL, TBL and any other small-group activities. If online group-work could be considered, that would be a bonus).One small issue:• “After World War II, large numbers of veterans furthered their educations and universities created make-shift student housing.” As this phenomenon did not occur world-wide, the location (USA?) should be identified.So, a useful read, but I would like to see Version 2 of the paper that applies the theory to medical education settings.

Reviewer Expertise:

NA

No decision status is available

# Reviewer report: Reviewer response for version 1

**DOI:** 10.21956/mep.19011.r27279

**Published:** 2021-01-13

**Authors:** Megan Anakin

**Affiliations:** 1 University of Otago

This review has been migrated. The reviewer awarded 3 stars out of 5 I read this article with interest because I am often apply concepts from sociology to understand phenomena in my health professional education research projects. I was curious to read about how you might suggest that we could apply social psychology principles to improve healthcare team settings and how these ideas could be incorporated into medical education programmes. I would be interested in learning more about how the suggested principles might be evaluated or studied further so that evidence can be presented about how they have been used to optimise effectiveness of healthcare professional team and students learning in medical education programmes.To make this article even more accessible to medical educators, please consider adding an example that is specifically related to medical education after the ‘applying principle to teams’ paragraph in each section. Please discuss how each principle might be turned into a learning opportunity in a clinical settings and suggest which teaching and learning methods might best put the principle into practice. Please state the benefits and challenges learners and teachers might encounter.I would be very happy to read and review a revised version of this article.

Reviewer Expertise:

NA

No decision status is available
